# Current concepts of anti-EGFR targeting in metastatic colorectal cancer

**DOI:** 10.3389/fonc.2022.1048166

**Published:** 2022-11-17

**Authors:** Bernhard Doleschal, Andreas Petzer, Holger Rumpold

**Affiliations:** ^1^ Department of Internal Medicine I for Hematology With Stem Cell Transplantation, Hemostaseology and Medical Oncology, Ordensklinikum Linz, Linz, Austria; ^2^ Gastrointestinal Cancer Center, Ordensklinikum Linz, Linz, Austria; ^3^ Johannes Kepler University Linz, Medical Faculty, Linz, Austria

**Keywords:** Metastatic colorectal cancer, epidermal growth factor receptor, clonal evolution, liquid biopsy, systemic treatment, molecular oncology, maintenance

## Abstract

Anti-EGFR targeting is one of the key strategies in the treatment of metastatic colorectal cancer (mCRC). For almost two decades oncologists have struggled to implement EGFR antibodies in the mCRC continuum of care. Both sidedness and *RAS* mutational status rank high among the predictive factors for the clinical efficacy of EGFR inhibitors. A prospective phase III trial has recently confirmed that anti-EGFR targeting confers an overall survival benefit only in left sided *RAS-wildtype* tumors when given in first line. It is a matter of discussion if more clinical benefit can be reached by considering putative primary resistance mechanisms (e.g., *HER2, BRAF, PIK3CA*, etc.) at this early stage of treatment. The value of this procedure in daily routine clinical utility has not yet been clearly delineated. Re-exposure to EGFR antibodies becomes increasingly crucial in the disease journey of mCRC. Yet re- induction or re-challenge strategies have been problematic as they relied on mathematical models that described the timely decay of EGFR antibody resistant clones. The advent of liquid biopsy and the implementation of more accurate next-generation sequencing (NGS) based high throughput methods allows for tracing of EGFR resistant clones in real time. These displays the spatiotemporal heterogeneity of metastatic disease compared to the former standard radiographic assessment and re-biopsy. These techniques may move EGFR inhibition in mCRC into the area of precision medicine in order to apply EGFR antibodies with the increase or decrease of EGFR resistant clones. This review critically discusses established concepts of tackling the EGFR pathway in mCRC and provides insight into the growing field of liquid biopsy guided personalized approaches of EGFR inhibition in mCRC.

## 1 Introduction

Colorectal cancer (CRC) remains a significant issue in global health. According to a recent analysis comparing the global cancer burden in the last decade, CRC is one of the 5 main causes of cancer-related disability-adjusted life years and is ranked 2nd after lung cancer, overtaking stomach cancer in 2019 (1). While rising cases are being reported, especially among the younger population, CRC is still a disease of the elderly. About 70% of cases occur between 50 and 80 years of age, with a mean onset of disease at the age of 72 for men and 75 for women (2). According to the Surveillance, Epidemiology, and End Results (SEER) database, 20% of patients present with primary metastatic CRC (mCRC) and 40% with relapse after previously curative intended treatment. The long-term outcome of mCRC is still poor, with a 5-year survival rate below 20% (1, 3). The treatment repertoire is stratified according to predictive biological markers of the respective tumors to leverage individualized treatment concepts. This treatment armamentarium has recently become more diverse and increased in number. These include: Monoclonal antibodies targeting the epithelial growth factor receptor (EGFR), such as Cetuximab or Panitumumab; HER2-directed agents such as Trastuzumab deruxtecan; antiangiogenic agents targeting vascular endothelial growth factor (VEGF) signalling, such as Bevacizumab, Aflibercept and Ramucirumab; as well as the broad spectrum kinase inhibitor Regorafenib. For microsatellite instable (MSI) cases, checkpoint inhibitors have evolved as a valid choice. These new treatment options have improved overall survival (OS) of patients with mCRC from approximately 1 year in the era of single agent 5-fluorouracil (5-FU) to more than 3 years with currently available options (2). This remarkable increase in survival appears to be largely based on the outcome data of left-sided mCRC (4). Left-sided mCRC has a more favourable predictive biological profile with a lower incidence of *RAS* mutations (*RAS*-MT) (5). Both sidedness and *RAS* mutational status rank high among the predictive factors for the clinical efficacy of EGFR inhibitors. In-depth knowledge of the appropriate integration of EGFR inhibitors in the continuum of care is required to gain maximum survival time for mCRC patients. This review will critically assess established concepts of EGFR targeting in mCRC in light of new diagnostic tools in order to shape the application of EGFR inhibitors in future clinical practice.

### 1.1 The EGF-Receptor

The discovery of EGFR as an oncogene is closely related to the history of our modern understanding of cancer pathogenesis (6–8). Almost half a century ago an EGFR variant that lacked sequences in the N-terminal ectodomain was found to promote aberrant cellular signalling in the absence of a binding ligand, thus transforming cells into a malignant phenotype. Some years before the findings of Cohen et al. showed that EGF ligand-dependent signalling *via* EGFR stimulated cellular processes like growth, proliferation and deemed worthy of Nobel prize honours (9). In the era when cancer was seen merely as a misguided signalling network, tackling aberrant EGFR signalling became one of the first goals of targeted cancer research (6, 7, 10, 11).

Decades of research later, EGFR is known to be embedded in a family of cell membrane-tagged receptor tyrosine kinases, including HER2/c-neu (ERBB2), HER3 (ERBB3) and HER4 (ERBB4). They share a common structure: a single amino acid chain protein forms an extracellular ligand binding domain, a transmembrane domain for homodimerization or heterodimerization and a tyrosine kinase intracellular portion. Each domain can initiate and drive malignant signalling; the ectodomain through binding ligands, the transmembrane domain allows ligand-independent signalling through dimerization, and amino acid modifications of the intracellular domain enables signalling regardless of ligand binding (10–12). Immunohistochemical (IHC) studies confirmed the expression of members of the ERBB family in various types of tumors. The mode of action depended on the tumor type and the isotype of the ERBB receptor (7). In breast cancer homo/-heterodimerization of ERBB2 and ERBB3 is the main route, while lung cancer is linked to tyrosine receptor mutations in the intracellular domain of ERBB1. CRC lacks activating mutations in ERBB1 and it was initially assumed that only overexpression of the physiological normal wild-type ERBB1 conferred tumorigenic activity (13). Blocking EGFR-mediated signalling in CRC through the development of antibodies competing for the binding site of physiological activators seemed a promising approach (8). Gill and Goldstein generated a mouse chimeric monoclonal antibody (IgG1) known as Cetuximab, which binds to the domain III of the extracellular domain with high affinity (14, 15). It renders the EGFR receptor in an inactive state mitigating downstream signalling pathways. Furthermore, it promotes receptor internalization, subsequent degradation and finally receptor down-regulation (15). Cetuximab is immunogenic in about 5% of patients. Therefore, a full human antibody (IgG2) against EGFR has been developed by immunization of transgenic mice (XenoMouse) known as Panitumumab. The mode of action is similar to Cetuximab. Differences in the IgG subclass that favour antibody-dependent cellular cytotoxicity and complement mediated cytotoxicity for IgG1 subtype antibodies appear negligible (15, 16).

Clinical evidence for the efficacy of EGFR inhibitors in the treatment of mCRC arose initially from phase II trials in later lines. Notably, mCRC included in these trials had to be EGFR IHC positive. Saltz et al. reported a clinical benefit rate in approximately half of the patients treated with Cetuximab as monotherapy (17). Together with Irinotecan, the overall response rate (ORR) doubled (23% vs. 11%) compared to monotherapy, as shown in the famous BOND trial by Cunningham et al. that finally prompted clinical approval of the drug by the FDA (18). Similar results for Panitumumab were obtained in 2006 by Giusti et al. that also led to FDA approval of Panitumumab in EGFR-expressing advanced mCRC after failure of first line therapy (19).

Interestingly, the BOND trial failed to confirm EGFR expression as a predictive marker for Cetuximab. Moreover, Cetuximab seemed to function even when EGFR was absent as measured by IHC and having sensitivity of IHC assays and probable tumor heterogeneity in mind. Though EGFR expression/amplification is not considered as a prerequisite for mCRC to be suitable for EGFR blockade, EGFR amplification (only 1% of mCRC) can booster EGFR inhibitors to exceptional outcomes in patients with *RAS/BRAF*-wildtype (WT) mCRC (20). EGFR amplification, albeit of no practical clinical significance, is the only positive predictive marker indicating exaggerated response to EGFR blockade thus far. The overall prognostic relevance of EGFR overexpression as an independent variable remains contradictory (21, 22).

## 2 Relevant clinical resistance mechanisms

### 2.1 EGFR ectodomain mutations

EGFR ectodomain (EGFR-ECD) mutations (exon 12) that prevent antibody binding rarely occur as a primary resistance mechanism. They typically evolve as a secondary resistance mechanism under the evolutionary pressure of sustained EGFR blockade (23). EGFR-ECD mutations are responsible for up to 25% of resistance mutations after failure of EGFR inhibitors (24). Usually, these patients experience deeper and longer responses to EGFR inhibitors in contrast to *RAS* related resistance mechanisms (23). Mutations in the extracellular domain S492, G465, S464, V441 are most prevalent, responsible for approximately 14% of all secondary resistance causes (23). Special attention should be paid to the variant S492 that has been found in 16% of patients after Cetuximab exposure and only 1% after Panitumumab treatment (25). A re-challenge approach with Panitumumab to overcome Cetuximab resistance in these cases would be rational from a molecular perspective, but prospective studies are still lacking to evaluate the efficacy of Panitumumab in the EGFR p.S492R mutant population (26).

Due to a seminal paper in 2006 the focus of main resistance mechanisms shifted to EGFR downstream pathway components (27).

### 2.2 *RAS* mutation

The MAPK pathway is one of the major downstream effectors of EGFR-based signalling in addition to the *Pi3K/AKT/mTOR* and PLCγ. The MAPK pathway consists of consecutive activated molecules named *RAS/RAF/MEK/ERK* that drive cell proliferation and malignant transformation (7, 10). In the 1980s *RAS* was one of the first cloned (proto)oncogenes characterized by Weinberg (28). During normal physiological states it is tethered to the plasma membrane through posttranslational modification mediated by farnesyltransferase (FTase) and acts a GTPase shifting from off to on states and back again (binary switch). This process is mainly regulated by extrinsic guanine nucleotide exchange factors (GEF) such as son of sevenless homologue 1 (SOS1) for GDP-to-GTP transition, and GTPase activating proteins (GAP) such as neurofibromin for GTP hydrolysis. A prominent RAS intrinsic GTPase activity is most prominently described for KRAS G12C and gets therapeutically exploited.

Activating mutations in the *RAS* family - consisting mostly of the isoforms *KRAS, NRAS*, and *HRAS* - are found in more than 20% of human cancers. In mCRC about 40% of the cases harbor activating mutations in *KRAS*, predominantly in exon 2 codon 12 (70-80%). Mutations in *NRAS* account for about 5%, particularly in exon 3 codon 61 (60%). *HRAS* mutations are rare in mCRC, but more dominant in head and neck cancer and urinary tract cancer. Secondary acquired *RAS* mutations after EGFR blockade are more often present as atypical mutations, e.g., *KRAS* codon 61 or codon 146 (29). The prognostic value of the *RAS*-MT in mCRC remains controversial, while it evolved as the crucial predictive biomarker in the treatment of mCRC (30).

In 2006 Lievre et al. published data from only 30 patients with mCRC treated with Cetuximab; they screened for mutations in *KRAS, BRAF*, and *PIK3CA* and their correlation with the response to EGFR inhibition and interestingly, none of the tumors harboring a *KRAS* mutation responded (27). These results were confirmed by other smaller trials, a retrospective analysis of phase III trials a metanalysis according to *RAS* status, and a smaller last line trial with panitumumab (19). Altogether, these results changed the treatment landscape of mCRC profoundly (31). In 2009 the FDA changed the label for Cetuximab and Panitumumab and then mandated *KRAS* exon 2 testing as a prerequisite. In 2009 the PRIME trial (Panitumumab randomized trial in combination with chemotherapy for metastatic CRC to determine efficacy) was first to test *KRAS* exon 2 mutational status prospectively as a predictive biomarker for mCRC (30). Panitumumab prolonged PFS by 1,4 months and an OS benefit of 4,4 months was only observed in *KRAS* exon 2-WT patients. PRIME further evaluated the possible negative predictive value of additional isoforms and exons of *RAS* and clearly showed that any activating mutation in *KRAS* or *NRAS* predicted resistance to Panitumumab (32). Similar data were acquired from a retrospective analysis of the CHRYSTAL or OPUS trial with Cetuximab as an EGFR antibody (33). According to recent ASCO and ESMO guidelines, expanded *RAS* biomarker testing (*all-RAS*) is standard of care. *RAS* analysis should comprise at least *KRAS* exons 2,3,4 and *NRAS* exons 2,3,4. All-*RAS* testing enhanced ORR response rates to EGFR antibodies from 20% to over 40%.

The advent of deep sequencing techniques allowed the detection of small *RAS*-MT subclones in biopsied tumor samples. The size of these subclones is commonly expressed in percentage as the number of sequence reads of a specific DNA variant divided by the overall coverage at that locus (variant allele frequency; VAF%). It can be explained as a surrogate parameter of the proportion of DNA molecules in the tumor specimen harboring this specific variant (e.g., RAS mutation). It was retrospectively observed that tumors carrying small *RAS*-MT subclones still benefit from EGFR blockade. The optimal cut-off value for *RAS*-MT tumors responding to EGFR inhibitors was determined by the ULTRA study. Tumors carrying *RAS* mutations below 5% VAF detected by deep sequencing (ddPCR) are still sensitive to EGFR blockade (34).

#### 2.2.1 KRAS G12C

The prevalence of KRAS p.G12C mutation in mCRC is about 3-4% according to different cohorts.

Among KRAS mutated mCRC, KRAS G12C is associated with shorter OS compared to KRAS non-p.G12C tumors. Reasons are unclear, but might be attributed to differences in metabolism and resistance mechanisms (35).In addition to its prognostic impact, KRAS G12C is characterized by its unique biophysical properties, which render KRAS G12C as the first targetable member of the so far undruggable RAS family. First, an outstanding intrinsic GTPase activity allows the covalent binding of small molecule inhibitors (e.g., Sotorasib or Adagrasib) during the inactive GDP state of KRAS (G12C), thus arresting the KRAS GDP and abrogating downstream signaling. Second, during the GDP state a recently discovered SII pocket is transiently formed, thus leveraging covalent binding of small molecule inhibitors to the cysteine residue of KRAS G12C (36–38).

In contrast to the clinical efficacy of KRAS G12C inhibition in lung cancer, the benefit in mCRC is less pronounced. CodeBreaK100 revealed an ORR of only 9,7% and a mPFS of 4 months when Sotorasib was used as monotherapy. Adagrasib in the KRYSTAL-1 trial showed slightly better but similar results. Distinct signaling networks in lung and CRC might partly explain differences in clinical efficacy. Similar to BRAF V600E inhibition, targeting KRAS G12C and consecutive downregulation of MPAK pathway stimulates EGFR signaling *via* a negative feedback loop mechanism (39–41). This attenuates the efficacy of Sotorasib or Adagrasib and activates bypass mechanisms. This phenomenon is almost only observed in mCRC, reflecting its dominant dependency on EGFR signaling. To overcome these limitations a strategy resembling targeting BRAF V600E in mCRC has been tested. In the KRYSTAL-1 trial Adagrasib combined with Cetuximab more than doubled response rates, whereas mPFS was only slightly improved by one month. CodeBreaK101 investigating Sotorasib+Panitumumab presented with similar clinical efficacy. The role of these treatment combinations in daily routine practice has to be finetuned in further trials (42, 43).

### 2.3 BRAF mutation

*BRAF* lies downstream of *RAS* in the MAPK pathway. Usually, *BRAF* alterations are classified according to their mode of activation. Whereas Class III is like the WT counterpart dependent on upstream *RAS* activation, class II and III work on *RAS* independently. Class I acts as active monomers; Class II is still dimer dependent. *BRAF-V600E* is the most important representative of Class I and occurs in 8–10% of CRC - ranked 5th across all cancer subtypes after hairy cell leukemia, papillary thyroid cancer, melanoma and Langerhans cell histiocytosis. It confers an extremely hostile phenotype with poor prognosis in mCRC, resulting in a nearly two-fold increase in mortality compared to wild-type *BRAF* mCRC (median PFS in first-line about 6 months and median OS about 13 months) (44).

*BRAF-V600E* is associated with right-sided tumors (up to 20%), high grade histology, higher patient age, female sex, MLH1 hypermethylation, serrated adenoma pathway and predominantly peritoneal metastatic spread; notably, *BRAF-V600E* is mutually exclusive to *RAS* mutations (44).

Class II/III alterations occur in about 2% of mCRC and are often associated, contrary to *BRAF*-V600E, with younger age of onset, male sex, left-sided primaries and a better prognosis.

Concurrent *BRAF-*V600E and MLH1 hypermethylation confer a MSI phenotype that profits from checkpoint inhibition (45, 46). *BRAF-*V600E mutations in MSS mCRC are one of the most negative prognostic markers in mCRC. This is well established from phase III trials like CRYSTAL, OPUS, COIN and TRIBE. An assumed negative predictive value for the efficacy of EGFR antibody-based therapy still remains controversial. Two meta-analyses of RCTs conducted by Rowland et al. and Pietrantonio et al. attempted to address this topic. Rowland et al. found no statistically significant difference in OS and PFS between *RAS*-WT/*BRAF*-MT and *RAS*-WT/*BRAF*-WT tumors, abrogating the negative predictive role of *BRAF-V600E* mutation for the use of anti-EGFR monoclonal antibodies in *RAS*-WT mCRC (47). However, Pietrantonio et al. demonstrated a lack of benefit with anti-EGFR treatment in patients with *BRAF-*V600E mutated CRCs (48). This finding was further strengthened by Stinzing et al. in the prospective FIRE4.5 trial (49). Based on its *RAS*-independent mode of action class III, *BRAF* mutations seem to retain sensitivity to EGFR-targeting (23, 50).

In the current European daily clinical practice these controversial results are discussed by stating that only 7,5% of patients with the *BRAF*-V600E mutation receive an anti-EGFR-based chemo-doublette in the first-line (51). EGFR blockade remains of significant importance in second-line treatment. According to the BEACON trial, additional EGFR inhibition is paramount, as *BRAF-*V600E altered MAPK pathway signalling is not sufficiently abrogated by *BRAF* and *MEK* inhibitors. This pathophysiological variation is unique to mCRC compared to other entities, e.g., melanoma. In mCRC inhibition of MAPK pathway leads *via* adaptive feedback loops to reactivation of the EGFR and exaggerated EGFR based signaling, thereby overcoming previous inhibitory attempts (52).

### 2.4 Human epidermal growth factor 2

The search for optimized biomarkers predicting EGFR efficacy was first uncovered in 2011 in preclinical data and later in a retrospective analysis of HER2 amplification/overexpression as a potential negative predictive marker for Cetuximab (53–55). It was shown that Homo- or heterodimerization of HER2 leads to activation of downstream signalling networks largely shared by EGFR signalling, thereby bypassing EGFR-mediated growth inhibition.

HER2 was first established in gastric cancer in the ToGA trial in 2010, which overexpresses HER2 in up to 20% of cases as a clinically valuable target (56). In mCRC, alteration in HER2 accounts for only up to 5% of cases and appears to be enriched in *RAS*-WT CRC, accounting for up to 40% in cases showing resistance to EGFR base antibody therapies (57, 58).

According to results from Sartor-Bianchi et al. and Raghav et al., HER2 amplification implicates worse clinical outcomes in EGFR-based first line trials in terms of ORR, PFS or OS (59, 60). However, upfront testing of *KRAS*-WT CRC for HER2 amplification or mutation is currently not recommended in the ESMO or ASCO guidelines. Targeting HER2 in mCRC currently finds its place in second or later lines (58, 61). First line trials are currently recruiting. The therapeutic landscape will presumably be revolutionized with the advent of novel tyrosine kinase inhibitors and HER2- targeting antibody drug conjugates (62).

### 2.5 Sidedness

The impact of primary tumor location on OS in mCRC dates back to the 1990s and was observed in several subsequent trials afterward (63). However, no clinical consequence was drawn from this observation. In 2016 a retrospective analysis of the CALGB/SWOG 80405 trial at ASCO 2016 and a later metanalysis including CRYSTAL, PRIME, CALGB/SWOG 80405, PEAK, and FIRE-3 changed the way we treat mCRC comparable to the impact of *RAS* mutation (64, 65).

CALGB/SWOG 80405 showed an OS of 33,3 months for left vs. 19,4 months for right-sided tumors. Furthermore, although the primary endpoint of the trial comparing Bevacizumab versus Cetuximab based doublettes in *RAS*-WT tumors was negative, dividing the trial population according to right vs. left disease deciphered the impact of EGFR inhibition on mCRC. In left-sided tumor *RAS*-WT Cetuximab almost tripled OS (39,3 vs 13,6 months) compared to right-sided *RAS*-WT tumors. Additionally, left-sided tumor benefited significantly from the addition of Cetuximab compared to Bevacizumab in terms of OS (39,3 vs 32,6 month) and PFS (64).

Two metanalyses enforced findings from CALGB/SWOG 80405 and EGFR inhibition was found to be superior to chemo-doublette alone (pooled analysis of CRYSTAL and PRIME) as well as compared to chemo-doublette+Bevacizumab pooled analysis of PEAK, FIRE-3 and CALGB/SWOG 80405) in terms of PFS and OS, but only in left-sided tumors (65–67). Therefore, left-sided primary tumor localization evolved as a positive predictive biomarker for efficacy of EGFR inhibitor therapy in *RAS*-WT mCRC.

At the ASCO meeting in 2022 the PARADIGM trial was the first trial to prospectively test the superiority of Panitumumab vs. Bevacizumab in combination with FOLFOLX6 in all-*RAS*-WT and left-sided primary tumors. OS increased from 34,3 months in the Bevacizumab arm to an unpreceded 37,9 months in the Panitumumab arm, whereas PFS remained comparable. Furthermore, ORR and R0 resection rates were increased by Panitumumab (68).The superior OS despite similar PFS rate of EGFR blockade compared to VEGFR inhibition in left-sided *RAS* WT tumors might be explained by deeper and earlier responses, expressed as early tumor shrinkage (ETS) and depth of response (DpR), according to a retrospective analysis and additional mathematical modeling (69–71). The DEEPER trial (JACCRO CC-13) with DpR as the primary endpoint confirmed the superiority of Cetuximab over Bevacizumab in terms of early tumor dynamics (72).

The differential sensitivity of right vs. left sided RAS-WT mCRC to EGFR inhibition is based on a diverse biological background. Right vs. left colon can be considered almost as two organs differing in numerous ways, including embryological development, bacterial colonization, gene expression levels during cancer development. Right sided mCRC display more often a MSI phenotype and resistance mutations of *RAS*, *BRAF*, and *PIK3CA* are more prevalent. Left-sided mCRC is characterized by chromosomal aberrations, HER2 and EGFR overexpression and a consensus molecular subtype (CMS) 2 and CMS 4 gene-expression profile (5). These differences may only be partly linked to the altered sensitivity to EGFR inhibition. Sidedness remains a stand-alone predictive factor. This was most recently confirmed in ultraselected mCRC cases, where left sided mCRC still conferred greater clinical benefit to EGFR blockade despite excluding mCRC with rare or ultra-rare resistance alterations in the EGFR pathway (73).

### 2.6 Emerging resistance mechanisms

Quadruple-WT CRC, hyperselection, and ultraselection are terms that describe molecular enrichment strategies to improve the efficacy outcome of EGFR inhibition. In 2011 a large retrospective analysis by De Roock and a later prospective CAPRi-GOIM trial coined the term quadruple WT CRC, indicating wild-type *KRAS/NRAS/BRAF/PIK3CA* tumors. In these trials, the ORR (64.4%) and median progression free survival (mPFS; 11.3 months) compared with patients exhibiting a mutation in one of these genes (ORR 47.4% and mPFS 7.7 months) was markedly improved (74, 75). Hyperselection means further refinement provided by the PRESSING (PRimary rESiStance IN *RAS* and *BRAF* wild-type mCRC patients treated with anti-EGFR monoclonal antibodies) panel including *HER2* amplification/activating mutations, *MET* amplification, *NTRK, ROS1, ALK, RET* rearrangements, *PIK3CA* exon 20 mutations, *PTEN* inactivating mutations, *AKT1* mutations. PRESSING alterations were more prevalent in right-sided tumors and more often associated with MSI status. Efficacy was initially demonstrated in a small case control trial and was further investigated in an exploratory analysis of the VALENTINO trial (76, 77). PRESSING positive tumors had significantly lower ORR (59% vs. 75%), PFS (7.7 vs. 12.1 months) and OS (68.1 vs. 48.1% 2 year OS rate). The PRESSING panel served as a predictive marker only in left-sided tumors, while right-sided tumors could not be further differentiated.

A further level of granularity was introduced by the PRESSING2 panel, which in addition to the PRESSING panel included rare and ultrarare potential resistance alterations (i.e., *NF1* mutations/loss, *ARAF/KRAS* amplification, *MAP2K1/MAP2K2* and *MAP2K4* mutations, *IGF1R* amplification, *ERBB3* amplification/mutations, *FGFR2* amplification, *AKT1/2* amplification, MSI status and *POLE* exonuclease domain). This enrichment strategy is commonly referred as ultraselection. About 50% of the *RAS/BRAF*-WT population harbor PRESSING2 mutations. The ORR (79%) and PFS rates (13 months) were comparable to the results of the PRESSING panel in the VALENTINO trial. The mOS rate of 51.2 months in left-sided PRESSSING2 negative tumors is unprecedented so far, extending mOS compared to the recently reported PARADIGM trials by 13.3 months (73). The PRESSING2 panel predicted outcome predominantly in left-sided tumors, but in contrast to the earlier PRESSING panel also right-sided PRESSING2 negative tumors gained benefit from EGFR blockade. It is therefore tempting to speculate that a defined ultra-selected subset of right-sided tumor might benefit from EGFR blockade - a finding with potential practical clinical implications (73).

## 3 Treatment algorithms in first-line treatment

Choice of first-line therapy is key in optimizing long-term outcome in mCRC. Achieving deep responses and long-term remissions in first-line is the prerequisite for maintenance concepts, treatment breaks and oligometastatic concepts; in short, it is the basis for the continuum of care concept (78).

For optimal induction therapy, four parameters are crucial to know in every routine clinical practice: *RAS* mutation status, MSI status, *BRAF-*V600E status and primary tumor localization (79, 80).

The largest benefit from EGFR inhibition is derived for left-sided *RAS/BRAF-WT*/MSS tumors. A retrospective analysis of three first-line trials observed an OS benefit of Cetuximab or Panitumumab over Bevacizumab, which was recently prospectively confirmed by the PARADIGM trial. Notably, HR for OS in the major trials (CALGB, PEAK, FIRE-3 and PARADIGM) was consistently comparable. PFS of second line was also beneficial after EGFR-based first-line as shown in FIRE-3. The STRATEGIC trial prospectively compared EGFR followed by Bevacizumab vs. Bevacizumab post progression and a numerical benefit in OS was observed (81).. The fact that the PFS of first-line remained the same between EGFR inhibition with either Panitumumab or Cetuximab vs. Bevacizumab, but OS showed a huge difference, may be largely explained by ETS in EGFR-based therapies and lack of EGFR response after VEGFR (69, 71, 82, 83).

The value of chemo-intensification in left-sided RAS-WT/BRAF-WT tumors was answered by the TRIPLETE trial. No benefit was reported when the chemo backbone was complemented by a third agent. Therefore, the mainstay for treatment of left-sided *RAS-WT/BRAF*-WT tumors l is still a chemo-doublette+EGFR inhibitor (84).

The value of EGFR inhibitors in right-sided *RAS*/*BRAF*-WT tumors is still controversial. Both the FIRE-3 and PEAK trials showed detrimental effects on OS compared to Bevacizumab, while a meta-analysis accounted for favourable ORR for EGFR. Therefore, for right-sided tumors where an oligometastatic concept seems feasible, EGFR inhibitors might still be of value. Optimal treatment of *BRAF*-MT/*RAS*-WT mCRC remains challenging. According to the FIRE 4.5 trial Cetuximab has a negative impact on clinical outcome data compared to Bevacizumab (85).

In the elderly frail population de-escalation strategies sparing a chemotherapeutic component are highly recommended. The PANDA trial clearly demonstrated that Oxaliplatin can be left out without losing efficacy (86). Furthermore, it compared favourably to the long-existing standard Capecitabin/Bevacizumab in terms of ORR, while preserving PFS (87).

In second and later line settings EGFR antibodies have failed to demonstrate any OS benefit (88–90). In particular, the sequence VEGF first-line→EGFR second-line in *RAS*-WT patients should be avoided (91, 92).

## 4 Maintenance

Typically, chemo-doublette+EGFR inhibition serves as an induction treatment for 4-6 months, as ETS and DpR in first-line treatment are key to overall OS benefit. Post-induction approaches should aim to consolidate the efficacy of induction treatment, while minimizing side-effects and preserving quality of life (QoL).

Three different treatment options exist. First, induction therapy can be continued until progression. This is only recommended for Irinotecan-based chemo-backbone by ESMO, as prolonged Oxaliplatin has debilitating effects on QoL parameters. In part, the same may hold for Cetuximab or Panitumumab. Second is a combination of drug holiday and re-exposure to chemo-doublette+antibody after progression. Third is de-escalation including withdrawal of one or two components of the induction regime and escalation upon progression; this is optimal for active maintenance approaches.

Evidence that de-intensifying after successful induction is feasible is derived from several trials. MACCRO-2 trial (Cetuximab vs. FOLFOX+Cetuximab), NORDIC VII (Cetuximab vs. FLOX+Cetuximab), SAPPHIRE (5-FU+Panitumumab vs. FOLFOX Panitumumab) and ERMES (Cetuximab vs. FOLFIRI+Cetuximab) preserved efficacy, while reducing incidence of peripheral neuropathy or acneiform rash (93–96). Maintenance of monotherapy versus drug holidays was compared in COIN-B, PRODIGE 28-time UNICANCER (Cetuximab vs. observation) and FOCUS4-N (Capecitabin vs. observation) (97–99). PFS was improved in the active maintenance arm, whereas OS remained unaffected. Prospective phase II trials (VALENTINO, PANAMA) favoured the combination of Capecitabin/5-FU+EGFR inhibition (Panitumumab) over the respective single agent (Panitumumab or Capecitabin/5-FU) in terms of prolongation of PFS without compromising QoL parameters (100). Maintenance with Bevacizumab +/- 5-FU in *RAS*-WT tumor was investigated in MACBETH (Cetuximab vs. Bevacizumab) and in a retrospective analysis of the PEAK trial (101, 102). In PEAK the median PFS and median OS from the discontinuation of Oxaliplatin were 9,7 vs. 7 months and 33,5 vs 23,3 months in the 5-FU- Panitumumab arm compared to the 5-FU Bevacizumab arm (102).

To summarize these different approaches and often conflicting results, a recent metanalysis was performed and revealed a PFS and even OS benefit for continuation of an EGFR-based doublette or active maintenance with EGFR + 5+FU over 5-FU or EGFR inhibitor monotherapy or observation (103). These findings were confirmed by a further meta-analysis of a larger real-world cohort and individual patient data pooled observations from the PANAMA and VALENTINO trial (104–107).

Predictive markers for the benefit of maintenance concepts are scarce. It is tempting to speculate that patients with SD disease compared to responding tumors derive the greatest benefit from post-induction treatments concepts according to FOUS4-N and PANAMA data. Biologically, tumors with PR and CR after 4-6 months of induction almost always experienced a maximal tumor response; presumably by eradicating the EGFR-sensible clonal population (82, 108, 109). Tumors with SD might already confer partial resistance mechanism requiring prolonged treatment or new concepts in the future (83). It is likely that only liquid biopsy will uncover forthcoming resistance mechanism as dynamic biomarkers for maintenance treatment stratification. Novel maintenance approaches in the MODUL or FOCUS-4 trials already incorporated more sophisticated stratified concepts recognizing the molecular portrait of the tumor at the end of induction phase (e.g., HER2+EGFR blockade or BRAF inhibitor+Capecitabin+EGFR inhibitor) (110, 111).

The idea of maintenance mandates re-exposure to a full first line induction scheme after progression. Only two trials prospectively evaluated this strategy. First, the phase 2 COIN-B study randomized patients with *KRAS* exon 2- wild-type mCRC with to receive FOLFOX–Cetuximab for 12 weeks, followed by Cetuximab maintenance vs. observation and reintroduction of FOLFOX–Cetuximab at progressive disease. There was no difference observed among the maintenance and intermittent strategies in 10-month failure-free survival (52% vs. 50%, respectively), even if a trend towards a better post-induction PFS (5.8 vs. 3.1 months, respectively) and OS (22.2 vs. 16.8 months, respectively) was noted in favor of the maintenance treatment (88).

Second, the prospective randomized phase 2 PANAMA trial compared 5-FU/LV+Panitumumab vs. 5-FU/LV alone as maintenance strategies in *RAS*-WT mCRC. The PFS of maintenance therapy was significantly improved with 5-FU/LV+Panitumumab (8.8 vs. 5.7 months), with a trend towards better OS (28.7 vs. 25.7 months). It is remarkable that time to failure of strategy (TFS) was only prolonged by 18 days (112).

The value of maintenance therapy after EGFR first line was recently further questioned by the IMPROVE trial at the annual ASCO meeting in 2022. It compared a continuous vs an intermittent treatment strategy with a chemo-doublette combined with EGFR inhibition by Cetuximab and suggest a similar OS in both arms (113).

These results argue for a strategy that includes drug holidays. However, there are several issues regarding trial end points that have to be re-evaluated. Nevertheless, the concept of intermittent or continuous treatment in a biologically favourable population is highly interesting.

The weighting of potentially improving OS and/or other clinical outcome markers against the accumulation of side-effects which reduce QoL has led to intensive discussion of the applicability of maintenance strategies in clinical practice. These issues should be included in new trials.

## 5 Liquid biopsy

Evaluation of tumor dynamics in metastatic disease still commonly relies on regular CT scans every 8-12 weeks. In routine clinical practice a differential outcome of the observed lesions can often be documented, drawing a heterogeneous portrait concerning sensitivity to a certain systemic therapy. Therefore, more accurate modes of response assessment have been eagerly awaited that take into account the spatial and temporal heterogeneity of metastatic tumor burden. Liquid biopsy has the potential to overcome these aforementioned limitations (114).

Liquid biopsy summarizes different non-invasive techniques for detection or monitoring of cancer. Circulating tumor cells (CTCs), tumor derived exosomes and cell free DNA (cfDNA) fragments can be measured in various body excretions and in depth biological information that impacts prognosis and further therapeutic choices can be drawn from e.g., a simple blood sample. CTCs and exosomes may provide a more extended image of the tumor- besides DNA based genomic information, including exosomal microRNA’s as biomarkers for prognosis and drug sensitivity prediction or CTCs for xenografting and *in vivo* drug testing (115, 116). Unfortunately, they miss reasonable sensitivity for e.g., tumor genomic alterations in comparison to cfDNA and are therefore not ready for broader clinical application (116–118).

In blood plasma cfDNA consists typically of 140-170bp in length originating mostly from leukocytes. As early as 1949 it was shown that patients with tumors derive a higher plasma cfDNA concentration (119, 120). The portion of cfDNA derived from cancer is called ctDNA and is depicted as variant allele fractions (VAF) typically ranging from <0,1 to 10% or higher.

In mCRC, evidence was first drawn by the detection of cfDNA in blood or stool probes more than 30 years ago (121). Today, ctDNA is on the edge of emerging as a viable biomarker in daily routine practice. New innovations in molecular biology techniques pushed the way forward. In 1999 the group of Bert Vogelstein set a milestone with the invention of digital-PCR (122). It allowed accurate qualitative and quantitative measuring of mutations against background noise aiming at 0,01% VAF or even lower. Years later Vogelstein et al. also developed the first high throughput digital-PCR method called BEAMing (beads, emulsions, amplification and magnetics), which allowed the routine application in research questions (123). Nevertheless, despite providing a sufficient technical limit of detection (LOD), digital PCR platforms are hampered by the restricted number of mutations per assay that can be analyzed. Next generation sequencing (NGS) of ctDNA can overcome these limitations by allowing detection of an infinite number of alterations including ones unknown so far. By establishing molecular barcoding (MB) techniques, LODs that may equal or even surpass that of digital PCR seem feasible (124–127). MB technologies combined with hybridize-capture-based methods (SureSelect XT HS and HaloPlex (Agilent) or amplicon-based methods (QIAseq Targeted Panel (Qiagen), IonAmpliSeq HD (Thermo Fisher Scientific), Signatera (Natera) are already commercially available. In CRC detection of minimal residual disease (MRD) after surgery might become one the first broad clinical applications of MB-NGS techniques (Signatera).

In metastatic disease, monitoring the course of the disease by following the tides of various mutations harboring subclones has gained increasing attention. In 2008 the Vogelstein group was again the first to assess tumor dynamics in mCRC by measuring serial ctDNA (*APC, TP53, KRAS* mutations) compared to plasma biomarkers and radiographic evaluation (123). An early drop of ctDNA was later prospectively validated as an indicator of tumor response overtaking conventional staging modalities (128).

The ESMO precision medicine working group recently recommended for the use of ctDNA in daily routine practice in chemotherapy-naive mCRC K*RAS*/N*RAS*/*BRAF*V600E/MSI testing by ctDNA if tissue testing is not feasible or urgent therapeutic decision making is necessary (129). During a metastatic disease course the ESMO precision medicine working group suggests ctDNA testing for *KRAS/NRAS/BRAF/EGFR-ECD/HER2* amplification. In contrast to tissue-based biopsy sampling of single lesions, ctDNA- based assays enables real-time portraits of tumor heterogeneity.

Concordance between *RAS* status in matched ctDNA and tumor tissue biopsy samples reaches over 90% according to various retrospective analyse and three larger prospectively conducted trials (130–137). For other mutations, concordance is quite similarly predictable, e.g., *BRAF*-V600E up to 100%, EGFR-ECD 99% (138). Reasons for impaired sensitivity of ctDNA plasma testing might depend on the specific ctDNA assay used, whereby OncoBEAM yielded the best results. On the other hand, ctDNA shedding seems to associate with specific tumor features. Low tumor burden, peritoneal and lung metastases and also mucinous histology hamper ctDNA release in contrast to high tumor burden and liver metastases (137).

Misale in 2012 pioneered the detection of acquired resistance to anti-EGFR therapy in mCRC. ctDNA of *KRAS* mutation or amplification were traced by BEAMing as early as 10 months before radiographic progression (139).

Resistance to EGFR inhibitor therapy is commonly associated with an alteration in the MAPK pathway. Emerging *RAS* mutated clones and EGFR-ECD mutations such as S492, G456, S464, V441 rank top among others like *MET, RAS*, and *HER2* amplification. Other genetic alterations that develop selectively under EGFR blockade involve *LRP1B, ZNF217, MAP2K1, PIK3CG, ATM, ATR, and BRCA1* (140). Furthermore, resistance mutations are by far not mutually exclusive. Rather, tumors with *KRAS* or EGFR mutations harbor >1 additional mutation in over 50%. Most data describing EGFR resistance mechanism are collected from EGFR application in later lines. Parseghian at ASCO 2021 demonstrated that results might be different in a strictly first line population conferring a rather low prevalence of so far established resistance mutations (141).

Resistance conferring clones are traced in plasma probes with a lead time of several months before clinical progress is visible on CT scans. Monitoring evolving clones occurring under EGFR inhibitor pressure allows earlier termination of ineffective therapies, enables strategies of continuation of EGFR blockade beyond progression and informs about potential targeted approaches, e.g., *MET* amplification- Crizotinib, HER2 amplification- T-DXd, *KRAS*-G12C mutation- Sotorasib, Adagrasib (142, 143).

Treatment with EGFR antibodies beyond progression commonly lacks valuable clinical implementation. Strategies to enrich All-RAS WT/BRAF- WT before second line EGFR blockade improved results formally but were not clinically meaningful. Resistance alteration besides RAS status, for example due to *MET* amplification might be relevant (144–148).

In contrast to continued EGFR blockade, rechallenging initial *RAS*-WT mCRC tumors with EGFR inhibitors in 3^rd^ line or later after proper EGFR inhibitor free intervals, seems a valuable further direction, which gained broader clinical applicability through the use of liquid biopsy (**Table 1**). Santini et al. in 2012 were one of the first to prove retrospectively that re-exposure of initial *RAS*-WT tumors with Irinotecan+Cetuximab reached PFS and OS benefit only in patients who were *RAS*-WT on ctDNA before re-challenge (151). However, the optimal intervening time interval between two EGFR inhibitor-based therapies remained a matter of debate. Aided by serial ctDNA measurements, Parseghian et al. calculated an exponential decay of *RAS* mutant or EGFR-ECD with a half-life of about 4 months. They also showed that, although not significant, the use of EGFR inhibitors after a treatment-free interval of at least 2 half-life cycles yielded the greatest ORR. The biological rationale for EGFR re-challenge is based on emerging and dwindling of pre-existing or acquired resistance conferring subclones. *RAS*-MT subclones pre-exist from the beginning and selective EGFR inhibitor pressure on *RAS*-WT clones leverages outgrowth of the *RAS-* MT clones to become the dominant one. Mutations in EGFR-ECD clonal populations are believed to occur as secondary events. The time course of other secondary resistance mutations is less well characterized.

**Table 1:** Trials investigating re-challenge strategies either with or without ctDNA guidance.

First author	Trial design	Patient sample	Outcome
**A. non ctDNA guided**			
**Schulz et al. (** 149)	Retrospective	21pts1 anti-EGFR free therapy	Rechallenge mPFS: 3,68month (m) mOS: 52,4m
**Parseghian et al. (** 150)	Retrospective cohort	80pts 5,1month (m) anti-EGFR free interval	ORR: 23% mPFS: 3,1m PFS and ORR higher depending on time interval
**Santini et al. (** 151)	Retrospective single arm, multicentre	39pts 6m anti-EGFR free interval	ORR 53% PFS: 6,6m
**Liu et al. (** 152)	Retrospective analysis of phase I/II trials	89pts 4,5m anti-EGFR free interval	mPFS: 4,9m; responders 1^st^ line
**Rossini et al. (** 153)	Retrospective real world, multicentre	86pts.	ORR: 19,8% mPFS: 3,8m mOS: 10,2m efficacy outcome assoc. with anti-EGFR free interval and lines of therapy
**Tsuji,** **JACCRO CC-09 trial** (154)	Prospective, multicentre phase II	25pts	ORR: 8,3% Efficacy dependent of anti-EGFR free interval (EFI) long EFI (1y) vs short EFI mPFS: 4,4m vs 2,5m mOS: 15,8m vs. 7,3m
**Tanioka et al.** (155)	Retrospective	14pts 13,1m interval	ORR: 3% mPFS: 4,2m
**Chong et al.** (156)	Retrospective	22pts 13,5m interval	DCR: 45,4% mPFS: 4,1m mOS: 7,7m
**Karani et al.** (157)	Retrospective	17pts 1-2 lines of intervening therapy	ORR: 18% mPFS: 3,3m mOS: 8,4m
**Masuishi et al.** **JACCRO CC-08 trial** (158)	Retrospective	34pts	Efficacy dependent of anti-EGFR free interval (EFI) long EFI (1y) vs short EFI mPFS: 4,6m vs. 2,1m mOS: 14,6m vs. 6,3m
**B. ctDNA guided**			
**Martinelli et al.-** **CAVE trial** (159)	Prospective, phase II trial	Interval>4m, 48pts ctDNA-WT: *RAS/BRAF/EGFR-S492R* 19pts ctDNA-MT	ctDNA-WT: mPFS: 4,1m; mOS: 17,3m ctDNA-MT: mPFS: 3m; mOS: 10,4m
**Mariani et al.** (160)	Multicentre, retrospective	26pts ctDNA WT: *RAS/BRAF* before rechallenge	ORR 25% mPFS: 3,5m mOS: 5m efficacy dependent on prev. response to EGFR inhibition, anti-EGFR free interval, previous lines of therapies.
**Cremolini et al.- CRICKET trial** (161)	Multicentre, phase II trial	13pts *RAS*-WT ctDNA 12pts *RAS*-MT ctDNA ctDNA *RAS/BRAF* status before rechallenge 4m interval at least	RAS ctDNA-WT vs ctDNA-MT ORR: 31% vs 0% mPFS: 4m vs 1,9m mOS: 12.5m vs 5,2m
**Sunakawa et al.** (162)	Retrospective	10pts *RAS*-WT ctDNA 6pts *RAS*-MT ctDNA ctDNA guided analysis of JACCRO CC-08/-09	RAS ctDNA-WT vs RAS ctDNA-MT DCR: 80% vs 33,3% mPFS: 4,7m vs 2,3m mOS: 16m vs 3,8m
**Sartori-Bianchi et al.- CHRONOS trial** (163)	Prospective	27pts, ctDNA-WT: *RAS, BRAF, EGFR-ECD* median 11,5m interval	ORR: 30% mPFS: 16weeks mOS: 55 weeks

Until now it remains unknown if the main reason for the decay of the resistant subclones is due to the effectiveness of EGFR free treatments or if predominantly the *RAS* WT clone gains growth advantage in relation to the *RAS*-MT clone, expressed in VAF% of total ctDNA.

Recently, several phase II trials such as REMARRY and PURSUIT, CRICKET (re-challenge Irinotecan+Cetuximab) or CAVE (re-challenge Avelumab+Cetuximab) confirmed these findings in larger, more defined cohorts. Inclusion criteria for CRICKET and CAVE required at least partial remission for 6 months during first-line EGFR inhibitor therapy, an intervening second line therapy and an EGFR treatment-free interval of at least 4 months. CRICKET reported an ORR of 21% and DCR of 54% in the ITT population (28 mCRC patients) (161). Baseline ctDNA testing was performed before re-challenge in 25 patients. 13 had *RAS*-WT and 12 *RAS-*MT ctDNA. In these 13 patients mOS and mPFS were 12.5 and 4 months, respectively whereas mPFS and mOS in the 12 patients with *RAS-*MT ctDNA were 1.9 and 5.2 months, respectively. ORR was extended to more than 30% of WT-ctDNA versus ITT.

In CAVE the primary end point was accomplished with mOS of 11.6 months (159). Disease control was 65% with a mPFS of 3.6 months. A significant difference in mOS was observed in patients with *RAS-*WT*/BRAF-*WT ctDNA at baseline compared to patients with mutated ctDNA (17.3 vs 10.4 months). Patients with mutated ctDNA reached a mPFS of 3.0 months whereas patients with *RAS-WT*/*BRAF-*WT ctDNA reached am PFS longer than 6 months in 41% of cases. ORR was only slightly enhanced from 8% (ITT) to 9% (*RAS-WT*/*BRAF*-WT ctDNA), but almost doubled compared to *RAS* or *BRAF* -MT ctDNA. The striking differences in ORR and mOS are most probably due to the EGFR partner: Chemotherapy vs. checkpoint inhibitor.

The recently published phase II CHRONOS trial pursued an anti-EGFR re-challenge strategy based on an interventional assessment of *RAS*, *BRAF* and EGFR-ECD status in ctDNA (163). It acknowledged the fact that the decay of resistance conferring subclones follows an individual timeframe, challenging a previous recommendation to wait at least 4- 8 months - the equivalent of one to two mutant clone half-lives. In CHRONOS a *“zero mutation ctDNA triage” of RAS*, *BRAF* and EGFR-ECD had to be evident at the time of re-challenge when compared with the time of progression. Out of 52 patients, 16 (31%) harbored at least one mutation that conferred resistance to anti-EGFR therapy and were excluded. A total of 27 patients were finally enrolled. A 30% response rate compared favorable with the response rates of 8% (CAVE) and 21% (CRICKET). The median PFS was comparable with about 4 months. Remarkably, although the median time between the last dose of EGFR directed therapy and CHRONOS screening was 11.5 months, 17 patients received screening within 8 months after the last EGFR-based therapy. Ten of these 17 patients were ctDNA negative again and could be included. Four patients responded (ORR 40%), 5 had stable disease and 3 progressive disease. Despite these small numbers, CHRONOS clearly favors individualizing re-challenge intervals for the single patient, which can only be reliably performed by liquid biopsy. Further refinement was derived from the REMARRY and PURSUIT trial at ASCO 2022 (164). These trials were designed to determine the efficacy of re-challenge strategies based not only on the negative ctDNA *RAS* mutation status just before preexposure to EGFR, but also, in particular, on the specific resistance mutations after EGFR blockade during first line treatment. Only tumors that did not develop *RAS* mutations as resistance mechanism during first line therapy responded to EGFR blockade, though *RAS*-MT ctDNA had to be cleared before re-challenge. These findings were recently reaffirmed by Topham et al. showing that the decay times of various acquired resistance mutations follow a different time scale (140). *RAS* mutations present quite tenaciously in contrast to other resistance mutations conferring the MAPK pathway, e.g., EGFR-ECD, *BRAF* or *MAP2K1* (140). Although it is too early to draw any routine clinical reasoning from these observations, one may conclude that it is highly likely that patients benefit in terms of PFS and OS from re-challenge strategies. Decisions for maintenance/re-challenge treatment strategies including EGFR inhibition can be refined by ctDNA measurements, as patients must show a negative ctDNA status as a prerequisite. Additionally, clonal resistance history and the time interval between EGFR treatments are important and can be monitored by ctDNA measurements (165).

Application of extended NGS based panels that cover multiple variants of secondary resistance such as *MET/HER2* amplification and *PIK3CA* mutation could further enrich responses of re-challenge strategies (hyper- or ultraselection), although a minimal clone size value in % VAF determining resistance is still missing. For *RAS* mutation a cut-off of 5% was reported as a gross orientation; interestingly also small RA-MT subclones might affect response parameters depicting a significant linear correlation clone size and response to EGFR blockade (108).

For a broad clinical application, the implementation of re-challenge strategies is still hampered by the fact that prospective trials comparing EGFR re-exposure to phase III proven treatments like TAS-102 or Regorafenib are still lacking. It is a promising strategy for the future as, for example, a cross-trial comparison of the CHRONOS trial compared to aforementioned third line options revealed a doubling of PFS and OS. Various meta-analyses already regard re-challenging strategies as mandatory to exploit tumor vulnerabilities serving optimized continuum of care concepts (149, 166).

## Conclusion

One of the main concepts that has been validated over the past 20 years to extend survival in mCRC is blocking the EGF receptor and inhibition of pathogenic MAPK signalling. Despite long-term routine use of Cetuximab or Panitumumab in everyday clinical care, the dawning of sophisticated molecular techniques including digital PCR, NGS and molecular barcoding among othersshed new light on the putative clinically relevant resistance mechanism. Use of liquid biopsy in clinical practice will optimize incorporation of EGFR inhibitors in the continuum of care of mCRC.

## Author contributions

BD wrote the first draft of the manuscript and all subsequent drafts. HR provided extensive revisions. AP extensively proofread the manuscript and contributed valuable points for discussion. All authors contributed to the article and approved the final version of the manuscript.

## Funding

This publication was supported by the Johannes Kepler Open Access Publishing Fund to cover the costs of the publication.

## Conflict of interest

The authors declare that the research was conducted in the absence of any commercial or financial relationships that could be construed as a potential conflict of interest.

## Publisher’s note

All claims expressed in this article are solely those of the authors and do not necessarily represent those of their affiliated organizations, or those of the publisher, the editors and the reviewers. Any product that may be evaluated in this article, or claim that may be made by its manufacturer, is not guaranteed or endorsed by the publisher.

## Abbreviations

mCRC, Metastatic colorectal cancer; VEGF, Vascular endothelial growth factor; MSI , Microsatellite instable; EGFR, Epithelial growth factor receptor; OS, Overall survival; IHC, Immunohistochemical; PR, Partial remission; SD, Stable disease; PFS, Progression free survival; ORR, Overall response rate; VAF, Variant allele frequency; QoL, Quality of life; LOD, Level of detection; ETS, Early tumor shrinkage; DpR, Depthness of response; WT, Wilde-type; MT, Mutated- type; MAPK, Mitogen-activated protein kinase; ddPCR, Digital droplet PCR; EGFR-ECD, EGFR extracellular domain; CMS, Consensus molecular subtypes; MB, Molecular barcoding; CTC, Circulating tumor cells; cfDNA, Circulating free DNA; ctDNA, Circulating tumor DNA; NGS, Next generation sequencing
